# Ripk1 and haematopoiesis: a case for LUBAC and Ripk3

**DOI:** 10.1038/s41418-018-0135-2

**Published:** 2018-06-04

**Authors:** Alessandro Annibaldi, Pascal Meier

**Affiliations:** The Breast Cancer Now Toby Robins Research Centre, Institute of Cancer Research, Fulham Road, London, SW3 6JB UK

Although long recognized as a component of inflamed tissues, the potential role of cell death as an active component that contributes to tissue homeostasis, inflammation and disease pathogenesis has only recently gained attention [1]. The notion that cell death components are hard-wired into inflammatory signaling pathways indicates that such death components act as positive regulators of tissue homeostasis, enhancing the resilience of epithelia to tissue stress [1, 2]

**Fig. 1:**
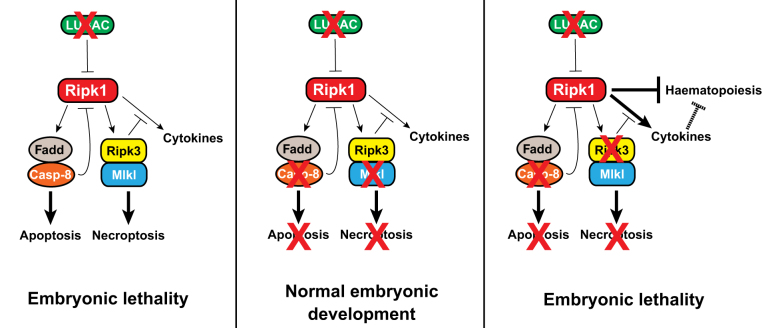
Models depicting LUBAC-mediated and Ripk-mediated regulation of embryonic development. The absence of LUBAC results in Ripk1/Ripk3/Caspase-8-dependent death of the embryo. Co-deletion of Caspase-8 and Mlkl restores embryonic development. However, when LUBAC, Caspase-8 and Ripk3 are mutated, Ripk1 impedes early haematopoiesis, possibly by driving ectopic cytokine production, leading to Ripk1-mediated embryonic lethality

Dedicated sensors have evolved to detect different stressors and induce adaptive responses. Receptor-interacting serine/threonine-protein kinase 1 (Ripk1) represents such sensor, functioning as signaling node in regulating adaptive responses to tissue stress [1]. Ripk1 is activated following a variety of input signals, such as in response to tumor necrosis factor (TNF) family cytokines, pattern recognition receptors and interferons. Ripk1 can activate mitogen-activated protein kinases, nuclear factor-κB (NF-κB) and apoptotic as well as necrotic cell death [1]. Ripk1 mediates these effects in a kinase-dependent and/or scaffold-dependent manner. While the scaffold function of Ripk1 maintains tissue homeostasis [1], the kinase activity of Ripk1 is required for necroptosis, and, depending on the cellular context, apoptosis [3, 4]. Ripk1 is tightly controlled by ubiquitylation, phosphorylation and caspase-mediated cleavage [1, 5, 6]. Too much or too little Ripk1 activity can lead to cell death, chronic inflammation and lethality. Understanding how Ripk1 is regulated has important implications for normal development and disease pathologies, such as chronic inflammation and cancer. But it also offers novel opportunities for immuno-oncology as Ripk1 and NF-κB signaling is required for antigen cross-priming of CD8+ T cells [7].

Much has been learned about Ripk1 regulation, and the pathophysiological consequences of its deregulation. In the current issue of *Nature*, Peltzer et al. [2] identified two new pieces of the puzzle. They discovered that the LUBAC component Hoil-1 prevents aberrant Ripk1-dependent cell death, and more unexpectedly, they uncovered a novel cell death-independent signaling output of Ripk1 that disrupts haematopoiesis in the developing embryo, and which is negatively regulated by LUBAC and Ripk3 (Fig. 1).

LUBAC is a tripartite E3 ligase complex composed of Hoip, Hoil-1 and Sharpin, capable of synthesizing linear ubiquitin chains [2]. LUBAC’s activity is required for optimal gene activation of pro-inflammatory cytokines. Interfering with LUBAC activity not only delays the gene activation program but also unleashes the cytotoxic potential of Ripk1 kinase. Genetic deletion of the LUBAC catalytic subunit *Hoip* causes embryonic lethality at mid-gestation due to TNF-mediated cell death of endothelial cells, leading to vasculature defects [8]. In the last issue of *Nature*, Peltzer et al. [9] expanded these studies and demonstrated that genetic deletion of *Hoil-1* also causes embryonic lethality at mid-gestation due to death of endothelial cells of the yolk sac. Although this phenocopies loss of *Hoip*, the lethality of *Hoil-1*-deficient mice was unexpected, first because Hoil-1 E3 ligase activity is dispensable for LUBAC activity *in vitro* [10], and second because Tokunaga et al. [11] previously showed that *Hoil-1*-deficient mice are viable. Peltzer et al. [9] demonstrated that while loss of Hoil-1 catalytic domain has no impact on LUBAC activity, the absence of the UBL domain thwarts LUBAC assembly and recruitment to the Tnfr1 signaling complex. Consequently, TNF stimulates Ripk1-mediated cell death in the absence of LUBAC.

The fact that *Ripk1* kinase-inactive mutant mice rescued the early lethality of *Hoil-1*-deficient mice is consistent with LUBAC being a negative regulator of Ripk1. However, this rescue was only partial. *Hoil-1*^*−/−*^embryos still died due to persistent endothelial cell death at late gestation. To address whether Ripk1 kinase-independent cell death could contribute to the lethality of *Hoil-1*^*−*^^*/−*^ embryos at late gestation, the authors co-deleted *Caspase-8* and *Ripk3* in *Hoil-1*-null mice to abrogate both apoptosis and necroptosis. Surprisingly, co-deletion of *Caspase-8 and Ripk3* did not confer any survival advantage when compared to Ripk1 kinase inactivation, although cell death was blocked. Even more surprisingly, co-deletion of *Caspase-8* and *Mlkl* allowed *Hoil-1*-null mice to be born and survive post the age of weaning. This unexpected difference between *Hoil-1*^-/-^*/Casp8*^−/^^−^*/Ripk3*^−/^^−^(lethal) and *Hoil-1*^-/-^*/Casp8*^−/^^−^*Mlkl*^−/^^−^ (viable) triple mutants prompted the authors to initially postulate that Mlkl might be activated independently of Ripk3 when LUBAC is nonfunctional. However, ablation of *Mlkl* in *Hoil-1*^*−/*^^*−*^*Casp8*^*−/*^^*−*^*Ripk3*^*−/*^^*−*^mice failed to prevent embryonic lethality. Therefore, the absence of Ripk3, rather than the presence of Mlkl, was detrimental to LUBAC mutant embryos. This led the authors to conclude that Ripk3 must possess a previously unrecognized, pro-survival function, which springs into action when both Hoil-1 and Caspase-8 are missing. Interestingly, *Hoil-1*^*−/*^^*−*^*Casp8*^*−/*^^*−*^*Ripk3*^*−/*^^*−*^ embryos had severe early haematopoietic defects, indicating that Ripk3 ensures proper haematopoiesis in the absence of LUBAC and Caspase-8. To elucidate the target of Ripk3’s pro-survival action, Peltzer et al. [2, 8, 9] generated a quadruple *Hoil-1*^*−/*^^*−*^*Caspase-8*^*−/*^^*−*^*Ripk3*^*−/*^^*−*^*Ripk1*^*−/*^^*−*^ mouse. This quadruple knockout mouse was indeed viable, suggesting that in the absence of Ripk3 and Hoil-1, deregulated Ripk1 signaling blocks early haematopoiesis, leading to embryonic lethality. Importantly, this lethal function seems to be independent of the kinase activity of Ripk1.

Therefore, Peltzer et al. [2, 8, 9] discovered a novel function for Ripk1. In the absence of Hoil-1 and Ripk3, Ripk1 acts as a negative regulator of haematopoiesis during embryonic development (Fig. 1). The fact that neither *Hoil-1*-null mice nor *Casp8*^*−/*^^*−*^*Ripk3*^*−/*^^*−*^ exhibit defects in haematopoiesis suggests that Hoil-1 and Ripk3, individually, are sufficient to suppress this function of Ripk1. While it is clear that the kinase activity of Ripk1 is not required for the new signaling role of Ripk1, it remains to be determined how Ripk1 impairs haematopoiesis via its scaffolding function. One possibility put forward by the authors is that deregulated Ripk1 drives ectopic cytokine production, which in turn interferes with haematopoiesis. In agreement with such a hypothesis, *Hoil-1*^*−/*^^*−*^*Casp8*^*−/*^^*−*^*Ripk3*^*−/*^^*−*^ animals harbour elevated levels of various cytokines (Fig. 1). This is also consistent with a recent report indicating that *Casp8*^*−/*^^*−*^*Ripk3*^*−/*^^*−*^ embryos exhibit aberrant Ripk1-mediated cytokine production, albeit this does not result in pathological changes [12]. According to this scenario, the high levels of cytokines of *Casp8*^*−/*^^*−*^*Ripk3*^*−/*^^*−*^ embryos might even further increase if *Hoil-1* is co-deleted, removing the last inhibitory stop on Ripk1. This might ultimately lead to pathological levels of cytokines that could impair haematopoiesis. Clearly, further work is needed to test this hypothesis. Moreover, it remains enigmatic how LUBAC suppresses Ripk1 activity in the absence of Ripk3 and Caspase-8. While LUBAC-mediated ubiquitylation of Ripk1 is possible, given that LUBAC also controls optimal activation of IKKβ and NF-κB-mediated gene expression, it is equally likely that LUBAC regulates Ripk1 activity indirectly, for example, in a phospho-dependent or transcription-dependent manner. Further, it remains unclear how Ripk1 is activated under these settings. Despite these open questions, the findings of Peltzer and colleagues clearly shed new light on the possible functions of Ripk1.

Given that Ripk1 and the production of damage associated molecular patterns (DAMPs) are required for antigen cross-priming of CD8+ T cells [7], a better understanding of Ripk1 regulation might also help to harness Ripk1 function for therapeutic purpose, particularly in the field of immunotherapy. Various cancer types loose expression of Ripk3 and/or acquire Caspase-8-inactivating mutations [13–15]. Therefore, inactivating LUBAC might drive Ripk1-mediated production of DAMPs. Combining LUBAC inhibition with cell death-inducing treatments might be a potentially effective approach to induce immunogenic cell death of cancer cells lacking Ripk3 and/or Caspase-8 activity. This might in turn mobilize the immune system against malignant cells and lead to durable anti-tumor immune responses.
